# A medical image encryption scheme based on Mobius transformation and Galois field

**DOI:** 10.1016/j.heliyon.2023.e23652

**Published:** 2023-12-12

**Authors:** Javed Ali, Muhammad Kamran Jamil, Amal S. Alali, Rashad Ali, Gulraiz

**Affiliations:** a Department of Mathematics, Riphah International University, Lahore, Pakistan; b Department of Mathematical Sciences, College of Science, Princess Nourah bint Abdulrehman University, P. O. Box 84428, Riyadh 11671, Saudi Arabia

**Keywords:** primary, 94A60, secondary, 68P25, Mobius transformation, Substitution box, Entropy, Correlation, Encryption

## Abstract

Data security and privacy are considered to be the biggest problems faced by service providers who have worked with public data for a long time. A key element of modern encryption that is utilized to increase textual confusion is the Substitution box (S-box) and the algebraic strength of the S-box has a significant impact on how secure the encryption method is. In this article, we present a unique method that uses a linear fractional transformation on a finite field to produce cryptographically robust S-boxes. Firstly, we choose a specific irreducible polynomial of degree 8 in $Z_{2}[x]$ to construct $GF(2^{8})$. Later, we used the action of $PGL(2,GF(2^{8}))$ on $GF(2^{8})$ to generate a robust S-box. The effectiveness of the built-in S-box was evaluated using several criteria including non-linearity, differential uniformity, strict avalanche criteria, linear approximation probability, and bit independence criterion. The proposed S-box's characteristics are compared to those of most recent S-boxes to confirm the higher performance. Additionally, the S box was used to encrypt images to show its usefulness for multimedia security applications. We performed several tests, including contrast, correlation, homogeneity, entropy, and energy, to evaluate the success of the encryption technique. The proposed method for ciphering an image is very effective, as proven by its comparison with several S boxes.

## 1 Introduction

The use of medical imaging to identify a variety of ailments has become widespread thanks to the quick advancements in medical device technology. Protecting medical images has become an increasingly important issue in recent years due to their transfer over multiple networks. Medical images require secure transfer with confidentiality, integrity, and authentication to protect patient data privacy. Additionally, if these images are susceptible to even the slightest modification, it could lead to an incorrect identification that endangers the lives of the patients. Some medical image-securing schemes are presented in [1], [2], [3], [4], [5], [6], [7]. One specific area of cryptography is symmetric cryptography which involves the use of stream and block ciphers. Some of the known block ciphers are Data Encryption Standard (DES) [8], International Data Encryption Algorithm (IDEA) [9], and Advanced Encryption Standard (AES) [10]. C.E. Shannon introduced the concept of block ciphers in 1949 [11]. In 1975, DES was introduced by IBM and subsequently adopted by the government of the USA for implementation in various sectors. However, in 1998 a group of university students decrypted the DES, causing the US government to seek proposals for a stronger encryption standard. The National Institute of Standards and Technology (NIST) accepted Rijndeal (AES) as the encryption standard and since then it has been used worldwide. AES uses keys with sizes of 128 bits, 192 bits, and 256 bits with 10, 12, and 14 rounds respectively. A round of AES has four steps: substitution byte, shift row, mix column, and add round key while the last round does not have Mix Column step. Due to the substitution box being the sole nonlinear part of AES, the block cipher's strength is largely determined by the strength of the associated substitution box. Thus, developing a sturdy S-box is a viable field of research. High non-linearity, resistance to linear and differential cryptanalysis, confusion, diffusion, bijectivity, algebraic complexity, and the lack of fixed points are all characteristics of a well-secured S-box. To construct efficient S-boxes, various techniques are utilized, including algebraic structures such as Galois fields, Galois rings, projective general and special linear groups, elliptic curves, coset diagrams, and Cayley graphs. It is intended to use an APA S-box which increases algebraic complexity by possessing the predicted accessible encryption features. A strong S-box was created by using the action of the Symmetric group of degree 8 on the original S-box of AES which is known as $S_{8}$ AES S-box. By performing several transformations on the AES S-box that were based on binary gray codes, the Gray S-box was constructed. The AES S-box, $S_{8}$ AES S-box, APA S-box, and Gray S-box are very strong S-boxes and are widely used in securing data transmission, [12], [13], [14], [15], [16], [17]. In addition to all of these, a variety of methods based on algebraic theory and chaotic theory were used to create substitution boxes. Several strong S-boxes have been proposed in the literature using various algebraic structures such as finite fields, rings, cyclic groups, symmetric groups, elliptic curves, and chain rings ([18], [19], [20], [21], [22], [23], [24], [25], [26], [27], [28], [29], [30], [31], [32], [33], [34], [35], [36], [37], [38], [39], [40], [47], [49]). As there are 30 irreducible polynomials of degree 8 over the field we can construct 30 fields of order 256. Although all of these fields are isomorphic, the strength of the substitution box might vary depending on the irreducible polynomials used and the boolean function selected. Almost all of the researchers used only specific irreducible polynomials $p(x)=x^{8}+x^{4}+x^{3}+x^{2}+1$ to design S-boxes and no discussion was made about cryptographic properties of other 29 irreducible polynomials. In this article, we selected a novel irreducible polynomial and used a specific Mobius transformation $\frac{kt+l}{mt+q}$ such that kq−lm≠0 and $k,q,l,m,t\in GF(2^{8})$ to generate the elements of S-box. It is important to assess the algebraic strength of the S-box before employing it in a cryptosystem because it is the only non-linear component of a block cipher. The algebraic strength of a substitution box is measured by various tests such as non-linearity, strict avalanche criteria, probability of linear approximation(LP), probability of differential approximation (DP), and bit independence criterion (BIC).

Six sections make up the remaining portion of the study. We covered a few fundamental concepts of the Galois field in Section 2. The algorithm for the construction of the S-box is found in Section 3. Many tests conducted on the recommended S-box and their comparison with other well-known S-boxes are covered in Section 4. In Section 5 we used the suggested S-box for image encryption and compared the results of the Majority Logic criterion with well-known S boxes. Finally, Section 6 concludes the study.

## 2 Galois field

A non-empty set *R* under two binary operation namely addition and multiplication is a ring if it is an abelian group under addition, semi-group with respect to multiplication and left, right distributive laws of multiplication over addition hold in *R*. Similar to the concept of normal subgroups in a group, an ideal in a ring is a subset *A* that satisfies two conditions: it is an additive subgroup of the ring and it is closed under left and right multiplication by elements of the ring. If the commutative law of multiplication holds in *R* then an ideal *A* of *R* is called maximal if A≠R and there is no other proper ideal of *R* containing *A*. A commutative ring *R* with unity which has no zero divisors is called an integral domain. Since $Z_{2}$ is a field, $Z_{2}[v]$ is an integral domain. Note that the polynomial $r(v)=v^{8}+v^{7}+v^{6}+v^{3}+v^{2}+v+1$ in $Z_{2}[v]$ is irreducible then the ideal <r(v)> is maximal in $Z_{2}[v]$ which implies that

$GF(2^{8})=\frac{Z_{2}[v]}{<r(v)>}=\{a_{7}t^{7}+a_{6}t^{6}+...a_{1}t+a_{0}|a_{0},a_{1},...,a_{7}\in Z_{2}\}$ is the Galois field of order 2^8^ where *t* is a particular root of r(v). Furthermore, ${\left(GF(2^{8})\right)}^{⁎}=GF(2^{8})-\{0\}$ is a cyclic group under multiplication and so we can write $GF(2^{8})=\{0\}\cup <v|v^{255}=1>$, here *v* is any element of order 255 in ${\left(GF(2^{8})\right)}^{⁎}$. Now the elements of $GF(2^{8})$ can be written in powers of *v* as follows

$$v^{0}=1=0\;v^{1}=v=1\;\;\vdots \;\vdots \;\vdots v^{7}=v^{7}=7\;v^{8}=v^{7}+v^{6}+v^{3}+v^{2}+v+1=763210\;v^{9}=v.v^{8}=v^{6}+v^{4}+1=640\;v^{10}=v.v^{9}=v^{7}+v^{5}+v=751\;\;\vdots \;\vdots \;\vdots v^{254}=v^{7}+v^{6}+v^{5}+v^{2}+v+1=765210\;0=\text{zero polynomial (additive identity)}$$

We can represent the elements of $Z_{256}$ in base 2 as follows

$$0=0\times 2^{7}+0\times 2^{6}+0\times 2^{5}+0\times 2^{4}+0\times 2^{3}+0\times 2^{2}+0\times 2^{1}+0\times 2^{0}\;1=0\times 2^{7}+0\times 2^{6}+0\times 2^{5}+0\times 2^{4}+0\times 2^{3}+0\times 2^{2}+0\times 2^{1}+1\times 2^{0}\;2=0\times 2^{7}+0\times 2^{6}+0\times 2^{5}+0\times 2^{4}+0\times 2^{3}+0\times 2^{2}+1\times 2^{1}+1\times 2^{0}\;\;\vdots \;\vdots \;\vdots 255=1\times 2^{7}+1\times 2^{6}+1\times 2^{5}+1\times 2^{4}+1\times 2^{3}+1\times 2^{2}+1\times 2^{1}+1\times 2^{0}$$

In order to destroy the structure of $GF(2^{8})$, we use the canonical bijection $Z_{256}\overset{2↔v}{↔}GF(256)$

Using this bijection we can express all the polynomials of $GF(2^{8})$ in the corresponding elements of $Z_{256}$ as well as in the power of *v* (see Table 1).

**Table 1:** Elements of *GF*(2^8^).

Decimal	Polynomial	Power	Decimal	Polynomial	Power	Decimal	Polynomial	power
1	0	0	2	1	1	3	10	141
4	2	2	5	20	27	6	21	142
7	210	162	8	3	3	9	30	48
10	31	28	11	310	61	12	32	143
13	320	132	14	321	163	15	3210	168
16	4	4	17	40	54	18	41	49
19	410	218	20	42	29	21	420	69
22	421	62	23	4210	18	24	43	144
25	430	241	26	431	133	27	4310	189
28	432	164	29	4320	202	30	4321	169
31	43210	206	32	5	5	33	50	92
34	51	55	35	510	89	36	52	50
37	520	251	38	521	219	39	5210	88
40	53	30	41	530	151	42	531	70
43	5310	127	44	532	63	45	5320	75
46	5321	19	47	53210	173	48	54	145
49	540	223	50	541	242	52	542	134
53	5420	104	54	5421	190	55	54210	213
55	54210	213	56	543	165	57	5430	159
58	5431	203	59	54310	15	60	5432	170
61	54320	85	62	54321	207	63	543210	210
64	6	6	65	60	96	66	61	93
67	610	235	68	62	56	69	620	122
70	621	40	71	6210	226	72	63	51
73	630	238	74	631	252	75	6310	45
76	632	220	77	6320	156	78	6321	89
79	63210	148	80	64	31	81	640	9
82	641	152	83	6410	109	84	642	71
85	6420	81	86	6421	128	87	64210	198
88	643	64	89	6430	99	90	6431	76
91	64310	182	92	6432	20	93	64320	113
94	64321	174	95	643210	245	96	65	146
97	650	43	98	651	224	99	6510	233
100	652	243	101	6520	180	102	6521	196
103	65210	107	104	653	135	105	6530	229
106	6531	105	107	65310	231	108	6532	191
109	65320	35	110	65321	214	111	653210	137
112	654	166	113	6540	59	114	6541	160
115	65410	139	116	6542	204	117	65420	187
118	65421	16	119	654210	216	120	6543	171
121	65430	125	122	65431	86	123	654310	37
124	65432	208	125	654320	13	126	654321	211
127	6543210	193	128	7	7	129	70	79
130	71	97	131	710	111	132	72	94
133	720	120	134	721	236	135	7210	154
136	73	57	137	730	85	138	731	123
139	7310	11	140	732	41	141	7320	178
142	7321	227	143	73210	33	144	74	52
145	740	67	146	741	239	147	7410	200
148	742	253	149	7420	25	150	7421	46
151	74210	130	152	743	221	153	7430	102
154	7431	157	155	74310	83	156	7432	90
157	74320	249	158	74321	149	159	743210	73
160	75	32	161	750	177	162	751	10
163	7510	184	164	752	153	165	7520	119S
166	7521	110	167	75210	78	168	753	72
169	7530	248	170	7531	82	171	75310	101
172	7532	129	173	75320	24	174	75321	199
175	753210	66	176	754	65	177	7540	23
178	7541	100	179	75410	247	180	7542	77
181	75420	118	182	75421	183	183	754210	176
184	7543	21	185	75430	116	186	75431	114
187	754310	115	188	75432	175	189	754320	117
190	754321	246	191	7543210	22	192	76	147
193	760	155	194	761	44	195	7610	237
196	762	225	197	7620	121	198	7621	234
199	76210	95	200	763	244	201	7630	112
202	7631	181	203	76310	98	204	7632	197
205	76320	80	206	76321	108	207	763210	8
208	764	136	209	7640	34	210	7641	230
211	76410	228	212	7642	106	213	76420	179
214	76421	232	215	764210	42	216	7643	192
217	76430	12	218	76431	36	219	764310	124
220	76432	215	221	764320	186	222	764321	138
223	7643210	58	224	765	167	225	7650	131
226	7651	60	227	76510	47	228	7652	161
229	76520	26	230	76521	140	231	765210	254
232	7653	205	233	76530	201	234	76531	188
235	765310	240	236	76532	17	237	765320	68
238	765321	217	239	7653210	53	240	7654	172
241	76540	74	242	76541	126	243	765410	150
244	76542	87	245	765420	250	246	765421	38
247	7654210	91	248	76543	209	249	765430	84
250	765431	14	251	7654310	158	252	765432	212
253	7654320	103	254	7654321	194	255	76543210	222

## 3 Algorithm for S-box

The map $\psi :\mathrm{PGL}(2,GF(2^{8}))\times GF(2^{8})\to GF(2^{8})$ defined by

$$\psi (\left[\begin{array}{cc} k & l\\ m & q \end{array}\right],t)=\left\{ \begin{array}{cc} \frac{kt+l}{mt+q} & :t\neq \frac{q}{m}\\ \frac{k}{m} & :t=\frac{q}{m} \end{array}\right.$$

where $k,l,m,q,t\in GF(2^{8})$ with kq−lm≠0 is a group action called Linear Fractional (Mobius) transformation.

It follows that for a fixed element $g=\left[\begin{array}{cc} k & l\\ m & p \end{array}\right]\in PGL(2,GF(2^{8}))$ the restriction $\psi_{g}:GF(2^{8})\to GF(2^{8})$ defined by $\psi_{g}(t)=\left\{ \begin{array}{cc} \frac{kt+l}{mt+q} & :t\neq \frac{q}{m}\\ \frac{k}{m} & :t=\frac{q}{m} \end{array}\right.$ is a bijection.

In particular we take k=29,l=115,m=81,q=19. In this way, kq−lm≠0 in $GF(2^{8})$, $\frac{q}{m}=248$ and $\frac{k}{m}=127$. The function $\psi_{g}(t)=\left\{ \begin{array}{cc} \frac{29t+115}{81t+19} & :t\neq \frac{19}{81}\\ \frac{29}{81} & :t=\frac{19}{81} \end{array}\right.$ reduces to $\psi_{g}(t)=\left\{ \begin{array}{cc} \frac{29t+115}{81t+19} & :t\neq 248\\ 127 & :t=248 \end{array}\right.$

Table 2 shows the working of algorithm, while Table 3 shows the outputs of *ψ* arranged as a 16×16 matrix.

**Table 2:** Algorithm for S-box.

*GF*(2^8^)	$\psi_{g}(t)=\left\{ \begin{array}{cc} \frac{29t+115}{81t+19} & :t\neq 248\\ 127 & :t=248 \end{array}\right.$	Element of S-box
0	$\psi_{g}(0)=\frac{29(0)+115}{81(0)+19}$	183
1	$\psi_{g}(1)=\frac{29(1)+115}{81(1)+19}$	197
2	$\psi_{g}(2)=\frac{29(2)+115}{81(2)+19}$	220
.	.	.
.	.	.
.	.	.
254	$\psi_{g}(254)=\frac{29(254)+115}{81(254)+19}$	59
255	$\psi_{g}(255)=\frac{29(255)+115}{81(255)+19}$	165

**Table 3:** S-box.

183	197	220	57	102	184	111	20	154	164	215	118	252	69	13	24
214	53	221	72	181	232	249	79	47	238	161	86	4	143	160	209
63	162	120	142	54	166	157	153	105	99	78	187	94	81	231	50
135	5	147	205	125	202	227	73	55	30	248	8	172	203	89	130
222	213	134	228	206	42	65	200	101	195	180	96	186	178	68	192
141	151	198	253	106	33	152	114	15	199	149	133	52	254	88	250
169	62	243	196	177	11	109	6	31	158	17	235	236	80	45	146
124	2	82	10	229	35	56	71	19	218	212	139	74	216	145	189
122	224	182	155	32	246	77	95	223	12	137	14	226	244	36	188
83	29	61	128	255	113	237	116	239	190	22	51	64	104	123	136
75	38	58	9	242	66	210	3	159	100	233	49	84	194	85	126
191	163	150	219	28	168	44	91	167	230	21	108	121	37	132	241
240	92	138	201	117	34	193	27	144	173	1	119	39	251	176	148
112	156	41	43	23	245	170	234	0	171	115	70	48	98	185	217
110	131	207	46	174	90	107	204	129	103	225	60	97	211	175	26
76	140	67	16	93	208	18	87	127	40	179	247	25	7	59	165

## 4 Algebraic analysis of the proposed S-box

To determine the suggested S-box's resistance to cryptographic assaults, security evaluations were conducted on it. The results are discussed in this section. The S-box was put through five different tests, including assessments for non-linearity, strict avalanche criteria (SAC), bit independence criteria (BIC), probability of linear approximation (LP), and probability of differential approximation (DP). The results of these evaluations were next contrasted with those of frequently employed S-boxes (Table 4).

**Table 4:** Algebraic analysis of proposed and some well-known S-boxes.

S-boxes	Nonlinearity	SAC	BIC-NL	BIC-SAC	LAP	DAP
Proposed S-box	112	0.4988	112	0.5008	0.0625	0.0156
AES	112	0.5049	112	0.5046	0.062	0.0156
[24]	105.5	0.507	106	0.462	0.140	0.0242
[25]	106.75	0.5032	103.6429	0.5074	0.1484	0.0469
*S*_8_ AES	112	0.504	112	0.502	0.062	0.0156
Skipjack	105.75	0.503	104.14	0.499	0.109	0.0468
Residue prime	99.5	0.515	101.71	0.502	0.132	0.281
[41]	110.25	0.50	104	0.5052	0.125	0.0390
[42]	109	0.5026	102	0.5026	0.1406	0.0390
[43]	109.25	0.5012	104	0.5056	0.0937	0.0312

### 4.1 Bijectiveness and balanacedness

An S-box is bijective if each input value is mapped to one and only one output value and each output value is derived from a unique input value. The decryption process in any cryptographic algorithm uses the inverse of S-box so a bijective S-box is an essential component for such algorithms. Moreover, a non-bijective S-box has weak cryptographic properties as compared to a bijective S-box. A boolean function is balanced if its truth table has an equal number of zeros and ones. An S-box is balanced if all of its component boolean functions are balanced. The security of a cryptographic technique using an imbalanced S-box can be compromised because its output distribution is biased towards specific bit values for some or all of its input values. Our proposed S-box is bijective and balanced. Moreover, it has no fixed points.

### 4.2 Nonlinearity (NL)

Nonlinearity is the quality of an S-box that deviates significantly from a linear connection between its input and output bits. In simpler terms, the output bits of an S-box cannot be predicted accurately by a simple linear combination of its input bits. Cryptography requires high nonlinearity in an S-box because it enhances system security by rendering it difficult for an attacker to deduce the input bits from the output bits. Various statistical tests, such as the correlation coefficient and Walsh transform, can measure nonlinearity. Our proposed S-box also achieves the optimal nonlinearity value of 112. The nonlinearity of an S-box can be calculated by the following

$$\mathrm{NL}(f)=\frac{2^{n}-max|{\mathrm{WHT}}_{f}|}{2},$$

where ${\mathrm{WHT}}_{f}$ is the Walsh Hadmard Transformation of *f*.

### 4.3 Strict avalanche criteria (SAC)

Strict avalanche criteria (SAC) [39] is a property that defines the behavior of an S-box in response to a small change in its input. It specifies that when a single bit of the input to an S-box is flipped, the output should change in an unpredictable and completely random manner with each output bit changing with a probability of exactly 0.5. In other words, the S-box should exhibit maximum diffusion, where any small change in the input propagates through the S-box, resulting in significant changes in the output. The SAC property is critical in cryptography as it ensures that any small modification in the input data results in a drastic transformation of the output making it challenging for attackers to decipher the original input data from the output data. If f(x)⊕f(x⊕c) is a balanced function for all *c* such that HWT(c)=1 then *f* satisfies the strict avalanche criterion.

### 4.4 Bit independence criteria (BIC)

Bit independence criteria for an S-box refers to a set of properties that specify the statistical independence between the input and output bits of an S-box. The criteria define the conditions that must be satisfied by the S-box to ensure that its output bits are statistically independent of its input bits. For the bit outputs $f_{i}$, $f_{j}(1\leq i,j\leq n,i\neq j)$ of an S-box, if $f_{i}⊕f_{j}$ is highly nonlinear and satisfies the strict avalanche criteria then the S-box fulfills BIC.

### 4.5 Linear approximation probability (LP)

The linear approximation probability of an S-box is a measure of the degree to which an S-box can be approximated by a linear function. In other words, it is a measure of the correlation between the input and output of an S-box when both are represented as binary vectors.

To compute the linear approximation probability of an S-box, we need to consider all possible pairs of input and output vectors and count the number of pairs that satisfy a specific linear equation. The linear approximation probability is then defined as the difference in the proportion of pairs that satisfy the linear equation with 0.5. S-boxes with low linear approximation probabilities are generally considered to be more secure against cryptographic attacks. LAP can be computed by the following equation

$$LP_{S}=\max_{u,v\neq 0}|\frac{|\{y\in GF(2^{n})\;|\;u.y=v.S(y)\}|-2^{n-1}}{2^{n}}|,$$

where u,v are the input and output masks respectively.

### 4.6 Differential approximation probability (DP)

The highest absolute difference between the occurrences of two separate input differences that lead to a given output difference across all conceivable input differences is known as the differential uniformity (DU) of an S-box. It measures the maximum difference between the probabilities of two input differences producing a certain output difference. This definition is useful for understanding how differential attacks work. Attackers look for input variations that enhance the probability of a specific output variation and these attacks can be thwarted by S-boxes with higher differential uniformity. The differential approximation probability (DP) is defined by

$$DP=\frac{DU}{2^{n}}$$

while DU is calculated by using

$$DU=|\{y\in GF(2^{n})\;|\;S(y)⊕S(v⊕\Delta y)=\Delta u\}|,$$

where Δ*y* is the input and Δ*u* is the output differential.

## 5 Application of proposed S-box in image encryption

In this section we will utilize the proposed S-box for encryption of medical images. We will check the effectiveness of image encryption algorithm by applying different tests on input image and cipher image.

### 5.1 Entropy

Entropy is a measurement of how chaotic or random the pixel values in an image are. Using Shannon's entropy formula, which takes into consideration the probability distribution of the various pixel values in the image, the entropy of an image may be computed. Entropy is calculated by the mathematical formula

$$\text{Entropy}=-\sum_{k}p(u_{k})log_{2}p(u_{k}).$$

When all 256 pixel values occur with the same probability an 8-bit gray scale image has an entropy value of 8 which is the greatest attainable value. Since the pixel values in a cipher image with an entropy value close to 8 are scattered as uniformly as possible, it is challenging to anticipate the original image from the cipher image.

### 5.2 Correlation

A mathematical procedure known as correlation involves swiping a filter over a picture and analyzing how similar the filter and the corresponding pixels in the image are. Correlation in the context of image encryption describes how similar the original and encrypted images are to one another. The strength of the encryption algorithm can be evaluated by looking at the correlation between the two images. An ideal correlation coefficient value for an image encryption system should be as close as 0. In practice, a correlation coefficient value of less than 0.1 is typically regarded as a reliable indicator of a high-quality encryption scheme, while a value greater than 0.1 denotes a possibility of weak encryption and the possibility of recovering the original image content from the encrypted image. The following formula can be used for calculating correlation;

$$r_{uv}=\frac{\sum_{i=1}^{m}(u_{i}-\overset{¯}{u})(v_{i}-\overset{¯}{v})}{\sqrt{\sum_{i=1}^{m}{\left(u_{i}-\overset{¯}{u}\right)}^{2}}\sqrt{\sum_{i=1}^{m}{\left(v_{i}-\overset{¯}{v}\right)}^{2}}}.$$

In this formula, *u* and *v* are the two images being compared, *m* is the number of pixels in the image.

### 5.3 Mean square error (MSE)

MSE is used to assess the quality of an image encryption protocol.

$$\mathrm{MSE}=\frac{1}{m\times n}\sum_{x=1}^{m}\sum_{y=1}^{n}{\left[O(x,y)-E(x,y)\right]}^{2}$$

The MSE value provides a quantitative measure of the distortion introduced by the encryption process. A lower MSE indicates less distortion and better encryption quality.

### 5.4 Peak signal to noise ratio (PSNR)

PSNR is also used to assess the efficacy of image encryption algorithms along with MSE. It quantifies the proportionality between the signal's maximum potential power and the power of the distortion introduced by the encryption process.

$$\text{PSNR}=10\cdot \log_{10}(\frac{{\text{M}}^{2}}{\sqrt{\mathrm{MSE}}}),$$

whereas *M* is the maximum pixel value. The PSNR value represents the quality of the encrypted image. Higher PSNR values indicate lower distortion and hence better encryption quality.

### 5.5 Key space analysis

**Figure 1:**
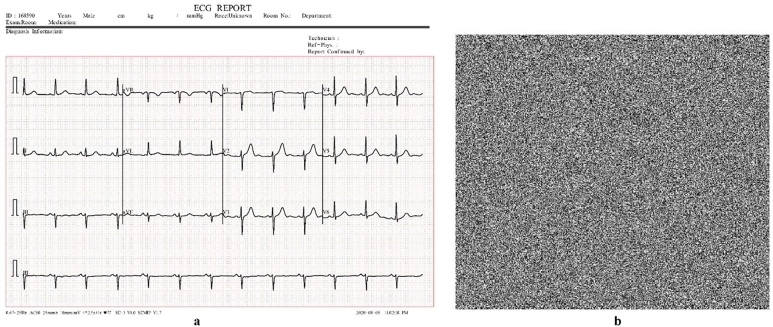
Plain and encrypted ECG image.

**Figure 2:**
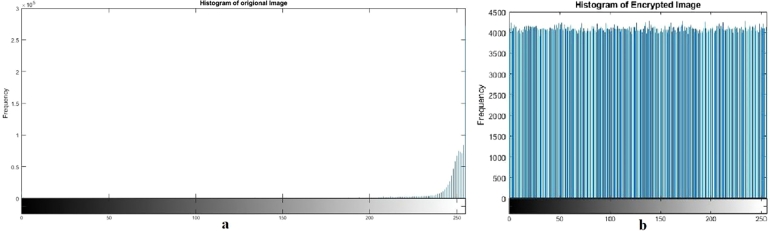
Histogram of plain and encrypted ECG image.

**Figure 3:**
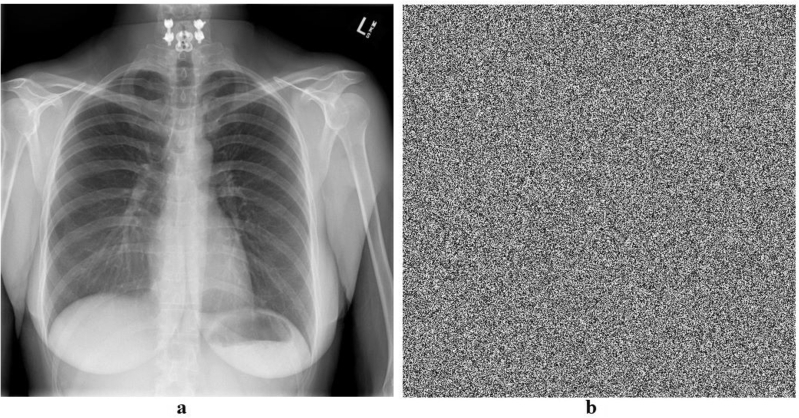
Plain and encrypted Xray image.

**Figure 4:**
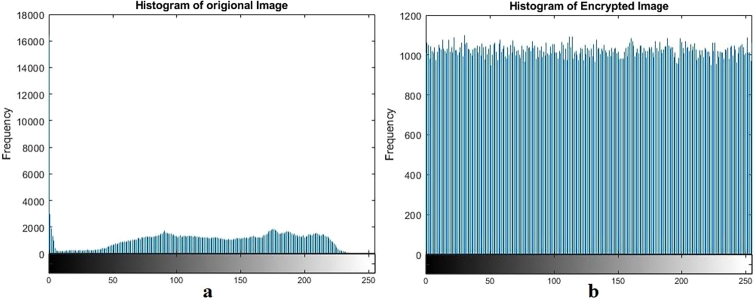
Histogram of plain and encrypted Xray image.

**Figure 5:**
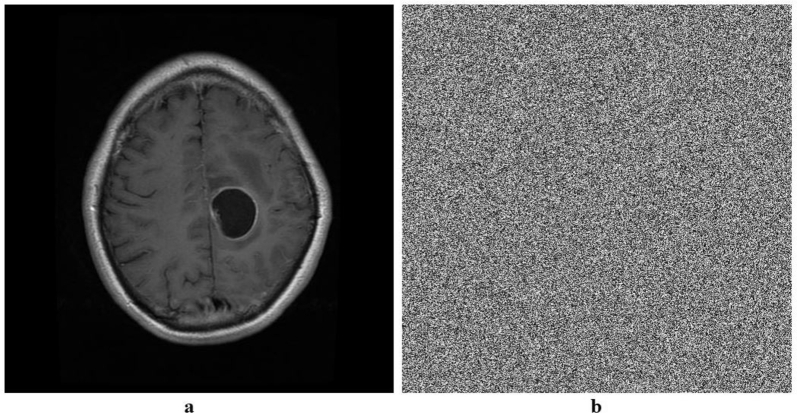
Plain and encrypted MRI image.

**Figure 6:**
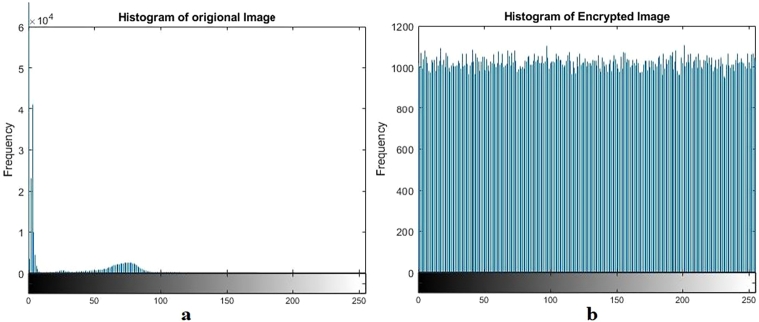
Histogram of plain and encrypted MRI image.

**Figure 7:**
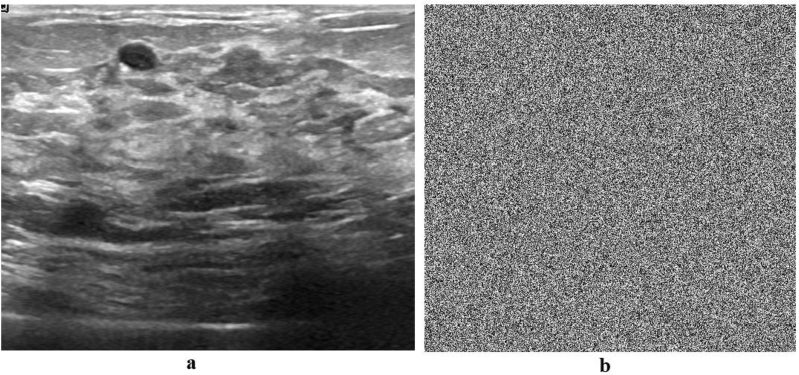
Plain and encrypted ultrasound image.

**Figure 8:**
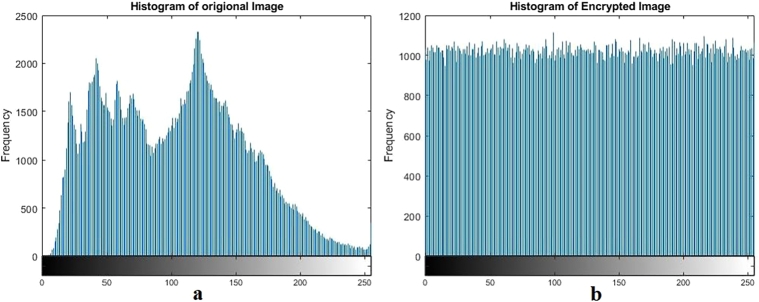
Histogram of plain and encrypted ultrasound image.

Keyspace analysis establishes the number of distinct keys that may be developed and employed. A larger key space is preferred since it increases the number of keys that an attacker must attempt in a brute-force assault to successfully decrypt data. Brute force attacks consist of repeatedly trying every key until the right one is discovered. This increases the safety of the encryption technique since a wider key space makes brute force attacks more computationally expensive and time-consuming. An image encryption scheme can withstand brute force attacks if its key space is at least 2^100^. The key space of our algorithm is 2^256^ (Table 5, Table 6, Figure 1, Figure 2, Figure 3, Figure 4, Figure 5, Figure 6, Figure 7, Figure 8).

**Table 5:** Results of different tests on image encryption scheme.

	Entropy	Correlation	NPCR	UACI	PSNR	MSE
X-Ray image	7.9994	-0.0061	99.60	33.49	28.20	9685.59
ECG image	7.9999	-0.0048	99.61	33.44	26.62	20045.73
Ultrasound image	7.9994	-0.0057	99.60	33.46	27	16835.5734
MRI image	7.9996	-0.0075	99.59	33.45	27.40	13946.48

Average	7.9996	-0.0060	99.60	33.46	27.31	15128.3433

**Table 6:** Comparative analysis of image encryption scheme.

	Entropy	Correlation	NPCR	UACI	PSNR	MSE
Proposed Scheme	7.9996	-0.0060	99.60	33.46	27.31	15128.3433
Ref. [44]	7.89	0.0194	99.80	33.29	5.7	780.12
Ref. [45]	-	-0.0074	99.66	33.50	-	-
Ref. [46]	7.9993	-0.0093	99.60	33.46	5.62	-
Ref. [48]	7.9985	-0.0055	99.94	33.57	7.18	12558.48

## 6 Conclusion

When used to encrypt medical images, the use of a new irreducible polynomial to build an S-box through the Mobius transformation has produced remarkable results that significantly outperform those of other encryption techniques. This accomplishment highlights the value of cutting-edge mathematical ideas and cryptographic methods in enhancing the security of sensitive medical data. Our method provides a dependable way to guarantee anonymity in healthcare by securely protecting patient information and diagnostics. It demonstrates the capability of cutting-edge research to address practical issues and increase data security, indicating improved prospects for the protection of medical and other sensitive data in our digital age.

## CRediT authorship contribution statement

**Javed Ali:** Conceptualization. **Muhammad Kamran Jamil:** Supervision. **Amal S. Alali:** Formal analysis. **Rashad Ali:** Visualization, Software. **Gulraiz:** Writing – review & editing, Writing – original draft.

## Declaration of Competing Interest

The authors declare that they have no known competing financial interests or personal relationships that could have appeared to influence the work reported in this paper.

## Data Availability

The findings of this study are not supported by any data.
